# How Nutrition Sensitive Are the Nutrition Policies of New Zealand Food Manufacturers? A Benchmarking Study

**DOI:** 10.3390/nu9121373

**Published:** 2017-12-19

**Authors:** Rebecca Doonan, Penny Field

**Affiliations:** Department of Human Nutrition, University of Otago, P.O. Box 56, Dunedin 9054, New Zealand; doore445@student.otago.ac.nz

**Keywords:** nutrition-sensitive, food, policy, scorecard, manufacturing

## Abstract

Nutrition sensitive policy addresses the underlying determinants of nutrition-related disease and is a powerful tool in reducing the incidence of non-communicable disease. Some members of the food industry have long standing commitments to health-oriented nutrition policies. The aim of this study was to develop and apply a balanced scorecard of nutrition sensitive indicators to the policies of influential New Zealand food and beverage manufacturers and explore factors affecting policy processes. Results: The average nutrition sensitivity score of the twenty influential manufacturers policies was 42 against a benchmark of 75. Some manufacturers performed well whilst others had substantial scope for improvement, the largest variation was in policy development and implementation, whereas nutrition quality was relatively consistent. Manufacturers with written policy (*n* = 11) scored on average three times higher than their counterparts with verbal policy. The value a manufacturer placed on nutrition influenced whether formal nutrition policies were developed. The reputational risk of failing to deliver on publicly declared nutrition commitments acted as an informal accountability mechanism. We conclude the balanced scorecard offers a useful tool for assessing the nutrition sensitivity of influential food and beverage manufacturers’ policies. Our results provide a baseline for repeat assessments of the nutrition sensitivity of food manufacturers’ policies.

## 1. Introduction

Scrutiny of food and beverage (F&B) company actions in relation to health reflects growing recognition of the critical role the private sector plays in tackling global nutrition issues [1,2,3]. With nearly two billion obese adults globally at an estimated cost of 2 trillion dollars annually, a range of effective strategies for reducing the risk of nutrition-related disease are urgently needed [1,2]. Policy stands out as a well-recognised, powerful, cost effective strategy for reversing some of the environmental drivers of nutrition-related disease [3,4,5,6,7]. 

In 2013 the World Health Organisation signalled the private sector’s responsibility for supporting and facilitating healthy lifestyles, with specific recommendations for the food industry [1]. For a number of years members of the food industry have attempted to demonstrate responsibility for the health of both their consumers and their staff [3,8]. Nutrition policies, including pledges and commitments, for activities such as marketing, staff health and product composition are increasingly common [9,10,11]. The nutrition community, however, generally remain sceptical of the motives and effectiveness of industry led and regulated policies [12,13]. A key concern is lack of independent monitoring and regulation by government or other organisations, transparency and accountability mechanisms [13,14,15,16,17]. Kraak et al.’s [14] distinction between responsibility and accountability encapsulates this concern. Responsibility refers to the acknowledgement of obligations from societal expectations, moral and/or legal standards, whereas accountability involves an individual or organisation answering to an empowered body. Nutritionists argue only independent bodies can objectively assess how well specific goals are achieved. Such organisations require authority to enforce policies to improve actions and outcomes [18]. 

To date there is limited comprehensive monitoring of food industry practices aimed at influencing health, beyond marketing activities. Two independent international groups, the Access to Nutrition Foundation [19] and the International Network for Food and Obesity/Non-communicable Diseases Research, Monitoring and Action Support (INFORMAS) [20], scrutinise differing food industry actions in relation to public health. Routine rating of the world’s 22–25 largest (based on sales) food manufacturers nutrition-related practices by the Access to Nutrition Index (ATNI) group hosted by the Access to Nutrition Foundation shows according to their criteria there have been some improvement, but in general industry is moving too slowly towards benchmark best practice and internationally agreed norms [19]. ATNI ranks companies nutrition-related practices across seven broad categories: governance, products, accessibility, marketing, lifestyle, labelling and engagement [19]. Whereas INFORMAS a global network of organisations and researchers aims to monitor, benchmark and support actions to create healthy food environments [20]. To date in New Zealand (NZ), INFORMAS has focused on expert ranking of government policies [21], and plans to assess food industry actions using a tool based on the ATNI. While monitoring by these groups provide valuable insight into food industry actions in relation to health, neither scrutinise the likely effectiveness of food companies policies. 

To be effective nutrition polices need to target the underlying determinants of poor nutrition including the availability (food supply), accessibility (affordability and allocation of food) and utilisation of nutritious foods [22,23]. The concept of ‘nutrition sensitivity’ comprehensively captures how well a policy addresses these determinants [22,24]. Nutrition sensitive policy has nutrition specific goals and actions aimed at developing health promoting environments for specific groups [22]. Distinct from nutrition policy, the concept of nutrition sensitive policy, adopted from agricultural policy in developing countries [25], offers a framework for focussing on the environmental drivers of nutrition related disease. Nutrition sensitivity has demonstrated its utility as a lens on public health nutrition policies, in particular evaluating school policy and developing benchmarks for assessing food environments [26,27].

As the food industry expand their commitments to health; governments, public health nutritionists and the food industry need a robust tool for benchmarking the effectiveness or ‘nutrition sensitivity’ of food companies’ policies. Balanced scorecards (BSC) provide a snapshot of organisational performance, whilst providing information on key areas to guide future direction [28,29]. Balanced scorecards have been regularly used by the commercial sector since Kaplan and Norton proposed deriving performance indicators from organisational visions and strategies across four ‘balanced’ dimensions (financial, customer, internal business process and learning and growth) [29]. More recently the BSC has been applied in the health sector, with the Commonwealth Fund [30] and Gauld et al. [28] developing and using a BSC to assess and compare health system performance in the United States and NZ respectively. Beaglehole and Bonita took a different approach using a BSC to assess global public health performance against four criteria for success [31]. From these few examples it is evident BSC’s offer a flexible tool for capturing organisational performance. Applying a BSC, with a range of weighted performance indicators, to organisational policy will generate a comprehensive assessment of performance. 

Along with other developed countries New Zealand (NZ) suffers heavily from the burden of nutrition-related non-communicable disease, and has the advantage of having readily identifiable influential food manufacturing companies [32]. Our study sought to determine the nutrition sensitivity of NZ F&B manufacturers’ policies by developing and applying a BSC of nutrition sensitive indicators. Two research questions guided our inquiry (a) how ‘nutrition sensitive’ are the policies of influential NZ F&B manufacturers; and (b) what factors influence manufacturers’ nutrition policy making. 

## 2. Materials and Methods

This research used a mixed methods, single case study design, undertaken in three phases, see Appendix A. A grounded theory approach [33] was used to evaluate the nutrition sensitivity of F&B manufacturers’ policies and to understand factors affecting the policy-making process. Mixed method approaches are widely used to analyse food and health policy as they allow consideration of a range of perspectives [33,34,35,36,37]. Phase one involved the development of a BSC of nutrition sensitive indicators, in phase two the researcher applied the BSC to manufacturers’ formal and informal nutrition policies, and in phase three semi-structured interviews were conducted with a company representative to explore factors affecting policy-making processes. 

### 2.1. Phase 1: Development of a Nutrition Sensitive Balanced Score Card

In developing a nutrition sensitive BSC we sought to bring together policy and nutrition indicators relevant to NZ F&B manufacturers’ policies. Policy indicators were derived from Walt and Gilson’s policy analysis triangle [38], a review of relevant food and nutrition policy literature and a recent BSC examining the nutrition sensitivity of NZ District Health Board policy [37,38,39,40]. Nutrition indicators were adapted from five of the seven ATNI categories: products, accessibility, marketing, lifestyles and labelling, to the NZ context [19]. For example, product labelling in NZ is governed by the Australia New Zealand Food Standards Code [41] so this indicator focused on uptake of the voluntary Health Star Rating system [42]. The remaining two ATNI categories, governance and engagement, were deemed to be captured in the BSC’s policy related indicators. The initial BSC was reviewed by two groups and pre-tested with three food industry experts. These experts have extensive experience working in the food industry, and were interested in collaborating with University researchers. The final scorecard featured 19 indicators across three dimensions: policy development, policy implementation and nutrition quality, outlined below in Table 1. 

Following Beaglehole and Bonita’s public health score card approach [31] each indicator was scored qualitatively. Four levels were used: not evident, emerging/developing, benchmark standard and exemplar. Benchmark criteria for each indicator were established from international or national guidelines or accepted best practice. Appendix B summarises the evidence used to formulate the benchmark for each indicator. To reflect the importance of policy processes in achieving nutrition outcomes, the scorecard was balanced with equal benchmark weighting attributed to policy development, policy implementation and nutrition quality. Within each dimension most indicators were weighted equally, however where the literature indicated a greater influence on F&B manufacturer’s policy a higher weighting was given, as shown in Table 1.

### 2.2. Phase 2: Application of the Balanced Scorecard to Manufacturers’ Policies

For each indicator manufacturers’ policies were subjectively scored against the evidence-informed benchmark of best practice. Manufacturers’ policies received an overall ‘nutrition sensitivity’ score relative to the total benchmark score of 75; manufacturer’s with exceptional policy in all three dimensions could score up to a maximum of 123 (exemplar level). 

Policy data was obtained by reviewing public websites, supplied internal nutrition-related policies and verbally provided policies. Each manufacturer was invited to review their preliminary BSC scores and send the researcher additional information to inform a review of scores. The researcher awarded a final score for each indicator based on professional judgment of all available data. 

### 2.3. Phase 3: Semi-Structured Phone Interview

To explore factors influencing the development, implementation and evaluation of nutrition sensitive policy, company representatives, with a role involving nutrition policy, took part in a 30-min semi-structured interview. Audio recorded interviews followed established qualitative interviewing and analysis protocols [43]. 

### 2.4. Recruitment

**Figure 1:**
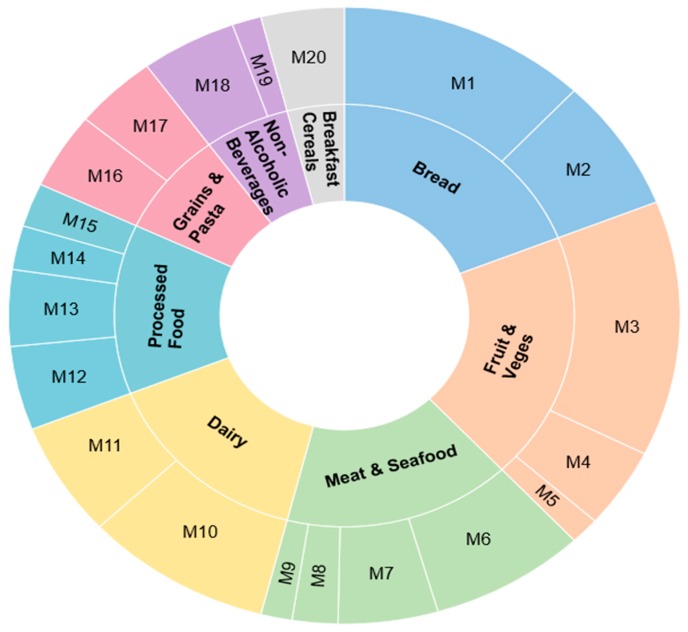
Main Energy Sources in the New Zealand (NZ) diet by food and beverage (F&B) Manufacturer Market Share. M: manufacturer.

Prior to recruitment, ethical approval was granted by the University of Otago Ethics Committee (reference number D16/264). To recruit manufacturers with the greatest influence on NZ food consumption, a novel approach to purposeful sampling was necessary. This involved reviewing and collating Adult Nutrition Survey data [44] to define the top eight sources of energy in the NZ diet. From this food manufacturers with the largest market share in each food category were identified using Euromonitor market share data [32]. Food retailers were outside the scope of this study. Recruitment was based on 20 manufacturers’ and their anonymity was protected to increase participation. Figure 1 presents the NZ market share of the anonymised manufacturers’ (M1–M20) for each of the major food groups. 

This figure presents the relative market share of manufacturers, for example there were multiple bread manufacturers, however, two companies dominated with M1 having a larger market share than M2. Two food industry experts reviewed identified manufacturers for their suitability and suggested four more manufacturers (M21–M24). 

Contact details were obtained for a company representative involved in nutrition policy for all twenty-four manufacturers. The BSC was applied to publically available and supplied policy documents of the twenty manufacturers who agreed to participate. Interviews were conducted with a representative of each manufacturer. Characteristics of participating manufacturers are presented in Table 2 below. Six of the twenty participating manufacturers were New Zealand owned, defined as equal to or greater than 50% New Zealand ownership and at least one manufacturer contributed to each major food category.

## 3. Results

**Figure 2:**
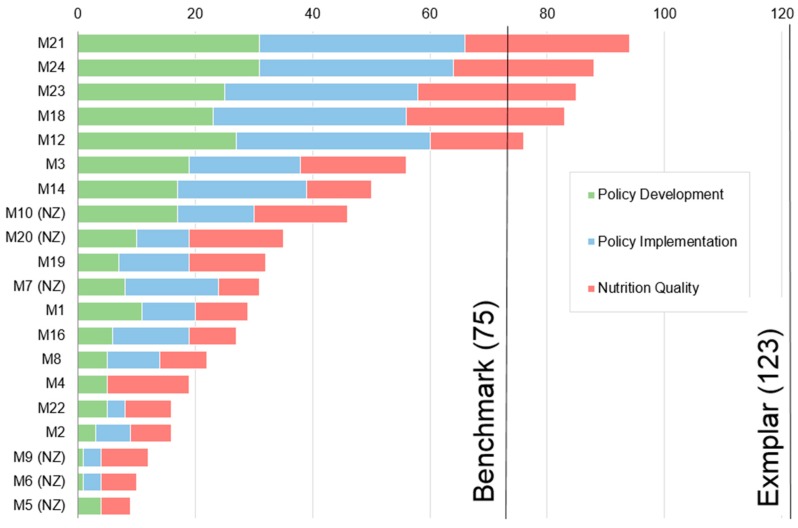
Manufacturer’s policies nutrition sensitivity scores.

The mean nutrition sensitivity score of all F&B manufacturers policies was 42, reflecting strong performance by some manufacturers and shortcomings by others. Total scores for each manufacturer by scorecard dimension are shown in Figure 2. 

Manufacturers’ mean score, maximum, 16th, 11th and 6th rank score and minimum for each dimension of the BSC, policy development, policy implementation and nutrition quality is shown in Table 3 below. Individual manufacturer’s scores for each indicator are presented in Appendix C.

The policy implementation dimension received the highest mean score, with some manufacturers performing exceptionally well, whilst others failing to implement any policy at all. In contrast, the nutrition quality dimension had the least variability as all manufacturers demonstrated some commitment towards nutrition, health and wellness as well as promoting aspects of nutrition or active lifestyles to consumers. The nutrition quality dimension also included the highest overall scoring indicator, ‘product formulation’, reflecting all manufacturers having at least considered reformulating or innovating products to improve their nutritional quality. The policy development dimension received the lowest mean score and contained the overall lowest scoring indicator, ‘accountability’. Most manufacturers (60%) had not even considered accountability when developing nutrition policy and none met the minimum benchmark level of having a clearly defined entity responsible for ensuring objectives are met and setting out the consequences of not achieving their objectives.

### 3.1. Impact of Policy Type

Total nutrition sensitivity scores differed widely between manufacturers with written nutrition policy and those with verbal policy, see Table 4 below. Manufacturers with written policy scored an average of three times higher in overall nutrition sensitivity than those with verbal policy with all nine overall top scoring manufacturers having written nutrition policy.

### 3.2. Impact of Country of Ownership

In general, overseas-owned manufacturers’ policies had higher overall nutrition sensitive scores than NZ owned manufacturers. Table 5 presents the mean BSC and dimension scores of NZ and overseas-owned manufacturers. Overseas-owned manufacturers mean nutrition sensitive scores were over twice as high as NZ owned manufacturers and achieved higher scores in each dimension. 

### 3.3. Factors Affecting Nutrition Sensitive Policy

**Figure 3:**
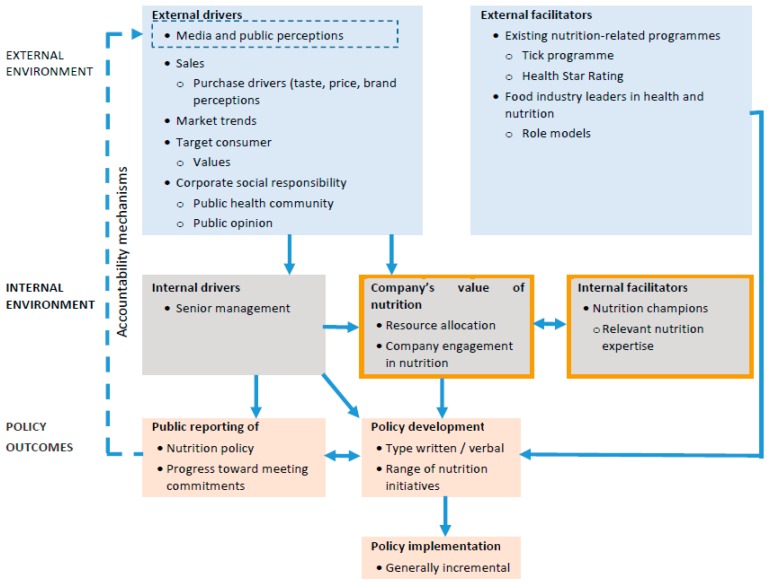
Factors influencing manufacturers’ development and implementation of nutrition sensitive policy.

Thematic analysis of interviews with twenty company employees revealed external and internal factors influence manufacturers’ development and implementation of nutrition sensitive policy. Figure 3 shows how interrelated factors in the internal and external manufacturers’ environment work with drivers and facilitators of nutrition sensitive policy. Drivers directly influence policy decision-making, whereas facilitators assist the process of developing and or implementing nutrition sensitive policy.

A strong theme emerging from the interviews was the priority or value placed on nutrition. A wide range of priorities regarding nutrition was evident when discussing product development. For a manufacturer focused on health and wellness “*nutrition is in our vision and everything is grounded in nutrition in some way, even if its confectionary*”, whereas for a manufacturer with a different focus “*first and foremost when we are designing products we design around taste*”. Despite the varying value placed on nutrition, most interviewees indicated an increase in general internal company awareness and openness around nutrition:

“*From a nutrition point of view I think there is an openness in asking whether we have got the opportunity to make changes*”

*M18*

To continue to be successful in highly competitive market places required continual review of external drivers. Manufacturers made decisions on business direction based on analysis of international market trends, customer demand and their own sale trends. In this context senior management exerted a major influence on how the company prioritises nutrition and how nutrition is incorporated into strategic plans:

“*The (nutrition) plan itself is something that is driven from the very top of our organisation and senior leadership really live and breathe it and that then filters down to everybody else*”

*M24*

Manufacturers who placed a high value on nutrition made it a company priority and were more willing to allocate resources to nutrition and invest in policy development. Nutrition champions, especially those with nutrition expertise were important facilitators in engaging internal stakeholders and the wider company in nutrition policy issues:

“…*There is a lot of dialogue going on “come on we can’t do this and we can’t do that”, and that is happening on a daily basis—we just have to really keep challenging our internal team and think creatively how else can we do it and just keep pushing.*”

*M11*

For most manufacturers nutrition-related actions occur incrementally over long periods of time. Sometimes the rationale was consumer benefit through stealth reformulations of popular products, other times it was to benefit company resources.

“*Enormous change doesn’t come in one big leap but actually in lots of little leaps, so its lots of steps creating a bigger shift in the long run*”

*M19*

For manufacturers with publicly declared nutrition commitments, interviewees acknowledged that the process of developing these commitments was resource intensive. Manufacturers wanted to make meaningful commitments that were achievable. Manufacturers believed they were under the microscope of the nutrition and public health community as well as consumers in relation to health and nutrition actions. Therefore, not delivering on public commitments represented a huge reputational risk. Resulting negative publicity, would adversely impact on public perception, so public commitments served as an informal accountability mechanism.

“*You are committing the company to do something and we know that if we don’t there will be a lot of bad media and reputational damage*”

*M21*

## 4. Discussion

The first ever scorecard on the nutrition sensitivity of NZ F&B manufacturers’ policies presents mixed results. It reflects a relatively good performance by some manufacturers and highlights substantial scope for improvement by others. The scorecard revealed large variations in performance in policy development and implementation, and smaller variation in nutrition quality. It is promising in that is demonstrates manufacturers’ are assuming some responsibility for health and making progress towards improving the health of staff and consumers. This resonates with evidence from the 2016 ATNI report showing global food manufacturers have taken steps since 2013 to improve consumers’ diets [19]. Some manufacturers’ were giving added weighting to nutrition in their corporate strategies’ while others were focusing on introducing more healthy options or improving labelling [19]. The current research reveals that in general, companies/manufacturers efforts are focused on developing and reformulating existing products to create more healthy options for consumers. 

Although the manufacturers in our study have undertaken positive steps it cannot be ignored that most manufacturers have serious work to do to increase the nutrition sensitivity of their policies. Overall our results are consistent with the latest ATNI findings [19] in showing that manufacturers’ with formal universally applicable policies, i.e., policies that apply across their product range, lead the way in nutrition sensitivity in comparison to those with informal policies or limited scope policies. These top performing manufacturers’ integrate nutrition into their corporate strategy as it contributes to their social responsibility obligations and, reflects commercial opportunities in increasingly nutrition conscious markets. Nutrition champions played an important role as internal change agents to help embed nutrition as a company value by engaging internal stakeholders. Nutley et al.’s observation on the influential role of intermediaries in the adoption of health sector policy appears to be highly applicable to food manufacturer’s adoption of nutrition policy [45]. 

Developing written policy is a resource intensive exercise and so is a good indicator of the value a company places on nutrition. The existence of written nutrition policy may also explain why higher overall scoring manufacturers performed decidedly better in policy development and implementation than lower scoring manufacturers. Often the deliberate processes required for writing policy compels policy makers to clarify their goals, consult stakeholders, consider evidence and balance competing priorities. The transparency and clear direction inherent in written policy is critical for effective policy implementation. Written policy also provides the clear statement of expected outcomes that Kraak argues are fundamental for external accountability [14]. 

The low scores for accountability across all manufacturers are likely to be of concern to the nutrition and public health community, who argue robust accountability mechanisms increase credibility and transparency of industry action, and are more likely to produce meaningful impacts on health [14,17,46]. Food industry self-regulation mechanisms are subject to criticism for not being sufficiently independent to meet the accountability standards of the nutrition and public health community [14]. These standards promote a shared responsibility model of accountability where an independent empowered organisation, such as government or a public health organisation, assesses company progress against intended outcomes and has authority to enforce policy change to improve outcomes [14]. In this regard, the nutrition sensitive BSC provides a practical tool for independent application of evidence informed benchmarks and easy identification of differences between companies as well as areas for improvement.

The benefit of the current BSC is that it focuses on nutrition sensitivity, i.e., the underlying determinants of nutrition, applying specifically to policy, a widely promoted strategy for tackling nutrition related non-communicable disease. The scorecard format offers a concise tool for comprehensively highlighting strengths and weaknesses of F&B manufacturers’ nutrition policies against evidence informed benchmarks that provide guidelines for improving policy. 

The current nutrition sensitive BSC is subject to limitations. First any scorecard is dependent on the source of data for analysis. Written, publicly available policy is ideal, however as some manufacturers lacked any written documentation in regards to nutrition, verbal information was taken at face value. Secondly, the scorecard was designed to assess the nutrition sensitivity of F&B manufacturers’ policies and was not able to distinguish whether this policy had any tangible impact on the food environment and population health. Thirdly, the scope of this BSC is limited to F&B manufacturers and to assess other important food industry actors, such as food retailers would require adaptions to the BSC.

## 5. Conclusions

These limitations aside, the scorecard has significant policy implications. Firstly the scorecard offers a comprehensive, simple tool for highlighting the strengths and weaknesses of F&B manufacturers’ nutrition policies in relation to achieving useful health outcomes. This current study provides a comprehensive, clear snapshot of current F&B manufacturers’ nutrition policies and benchmarks for tracking changes to the nutrition sensitivity of policies. Furthermore the BSC has the potential to be used internally by manufacturers’ as a guide to improving their own nutrition policies and externally by other key actors’ including nutritionists, public health communities and/or governments to strengthen accountability mechanisms.

## Figures and Tables

**Table 1:** Balanced scorecards (BSC) structure.

Dimension	Policy Development	Policy Implementation	Nutrition Quality
Indicators	Benchmark level 3	Benchmark level 5	Benchmark level 3
	Purpose Objective Responsibilities Scope Stakeholder Consultation Evidence Base Level of company commitment Accountability	Timeframe Communication Auditing Evaluation of policy from target group Access Auditing Access	Product formulation Marketing to all consumers Marketing to children Product accessibility Staff health and wellness Consumer-orientated healthy eating and lifestyle programmes
	Benchmark level 4		Benchmark level 5
	Accountability		Product formulation Product accessibility

**Table 2:** Manufacturers characteristics ^1,2^.

Manufacturers	Staff Employed	Revenue	Primary Country of Ownership
M10	16,000	$22,275 m	New Zealand
M1	1800	$939 m	Singapore, Hong Kong
M3	1600	$722 m	USA
M18	1100	$584 m	Australia
M8	2100	$517	Singapore
M7	1100	$448	New Zealand, Japan
M19	900	$438 m	Japan
M21	750	$390 m	Swiss
M12	500	$298 m	USA
M2	1000	$266 m	UK
M4	400–500	$257 m	Canada
M6	600+	$240 m	New Zealand
M23	350	194 m	USA
M22	420	$172	Australia
M20	350	$150 m	New Zealand
M10	120	$70–80 m	New Zealand
M16	2100	-	Australia
M5	11–20	-	New Zealand
M14	-	-	Australia
M24	2000	-	The Netherlands, UK

^1^ Reference not supplied to protect anonymity; ^2^ Captures revenue and staff employment in the New Zealand based branch of manufacturers and therefore may not be representative of a company globally.

**Table 3:** Manufacturers’ overall BSC scores and range by dimension.

Score	Policy Development	Policy Implementation	Nutrition Quality	Total
Benchmark level	25	25	25	75
Exemplar Level	41	39	43	123
Mean	13	15	14	42
Max	31	35	28	94
16th rank	27	33	16	76
11th rank	7	12	13	32
6th rank	5	0	14	19
Min	1	0	5	9

**Table 4:** Impact of policy type on the nutrition sensitivity scores of manufacturers policies.

Policy Type	Total Mean Score	Development	Implementation	Nutrition Quality
Benchmark Level	75	25	25	25
Written (*n* = 11)	61	20	23	18
Verbal (*n* = 9)	18	4	5	8

**Table 5:** Mean nutrition sensitivity scores of NZ and overseas owned manufacturers policies.

	Total Mean Score	Development	Implementation	Nutrition Quality
Benchmark Level	75	25	25	25
NZ ownership	32	7	7	10
Overseas Ownership	66	15	19	16

**Figure A1:**
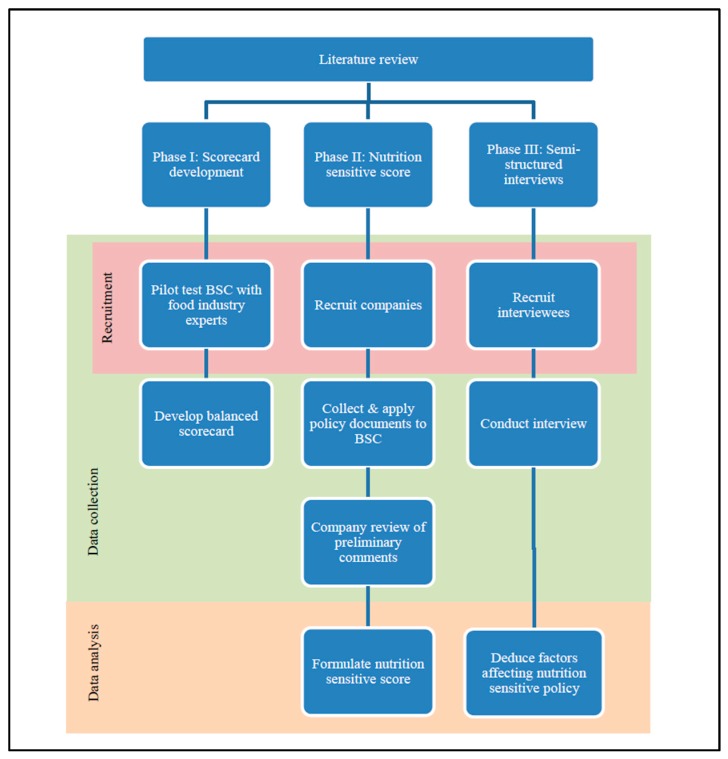
Three Phase Research Design.

## Appendix B

**Table A1:** Source of Evidence for BSC Indicators Benchmarks.

Indicator (s)	Source of Benchmark Evidence
**Policy development and framework**	
Purpose, objective, responsibilities, scope, stakeholder consultation, evidence base and level of company commitment.	Pyatt’s balanced scorecard [37]
Accountability	Kraak’s accountability framework [14]
**Policy implementation**	
Timeframe, communication, auditing, evaluation of policy from target group and access.	Pyatt’s balanced scorecard [37]
**Nutrition quality**	
Product formulation	For healthy product innovation, best practice is regarded as following recognised guidelines such as those published by Codex [47], WHO [3,48,49,50], FAO [51] and the New Zealand Ministry of Health [52].
Product accessibility, consumer orientated healthy eating programmes	Currently there is limited international norms or guidelines established in regards to product accessibility (price and distribution) or consumer-orientated healthy eating programmes. The benchmarks for these indicators was based off the ATNI framework [19]
Marketing to all consumers, marketing to children	Internationally there is a raft of voluntary guidelines and codes related to responsible advertising to children [9,10,53,54,55]. The most widely supported general marketing codes are produced by the International Chamber of Commerce, which form the basis of many national self-regulatory marketing codes [56], including the New Zealand Advertising Standards Authority codes [57].
Staff health and wellness	The 2016 US Chamber of Commerce report “Winning with Wellness” captures the core principles of an effective staff health and wellness programme [58]. The ATNI also produce a table of components of workplace health and wellness programmes [19].
Labelling	Codex provides international guidance on food labelling [47], but in the New Zealand context the government controlled Food Standards Australia New Zealand (FSANZ) mandatory food standard code covers nutrition labelling and has strict requirements for health and nutrition claims on products [41]. In New Zealand, the government endorsed Health Star Rating is a voluntary front-of-pack labelling schemes for New Zealand food manufacturers and was therefore the focus of this BSC. The benchmark was formulated with the assistance of a food industry expert.

## Appendix C. Tables of BSC by Dimension and Indicator Scores

**Table A2:** Policy Development and Framework.

Company	Total	Purpose	Objective	Responsibilities	Scope	Stake-Holder Consultation	Evidence Base	Company Commitment	Account-Ability
**Benchmark**	**25**	**3**	**3**	**3**	**3**	**3**	**3**	**3**	**4**
M1	11	1	1	3	3	1	1	1	0
M2	3	0	0	1	0	1	0	1	0
M3	19	3	3	1	3	3	3	1	2
M4	5	0	1	0	0	0	1	3	0
M5	4	0	0	0	1	0	0	3	0
M6	1	0	0	0	0	0	0	1	0
M7	8	3	0	1	3	0	0	1	0
M8	5	0	1	1	0	1	1	1	0
M9	1	0	0	0	0	0	0	1	0
M10	17	1	3	3	1	3	1	3	2
M12	27	3	5	3	3	3	5	3	2
M14	17	3	3	1	1	3	1	3	2
M16	6	0	1	1	0	1	0	3	0
M18	23	3	3	1	1	5	5	3	2
M19	7	0	3	1	0	1	1	1	0
M20	10	0	0	1	0	3	3	3	0
M21	31	3	3	3	5	5	5	5	2
M22	5	0	0	0	0	1	1	3	0
M23	25	3	5	1	3	3	5	3	2
M24	31	5	5	3	3	3	5	5	2
**Mean**	**12.8**	**1.4**	**1.9**	**1.2**	**1.4**	**1.9**	**1.9**	**2.4**	**0.8**

**Table A3:** Policy Implementation and Monitoring.

Company	Total	Timeframe	Communication	Auditing	Evaluation of Policy from Target Group	Access
**Benchmark**	**25**	**5**	**5**	**5**	**5**	**5**
M1	9	0	0	3	3	3
M2	6	3	0	3	0	0
M3	19	5	3	3	3	5
M4	0	0	0	0	0	0
M5	0	0	0	0	0	0
M6	3	0	3	0	0	0
M7	16	3	5	5	0	3
M8	9	3	0	3	3	0
M9	3	0	0	0	3	0
M10	13	0	0	5	3	5
M12	33	5	7	9	3	9
M14	22	5	5	7	0	5
M16	13	0	3	5	0	5
M18	33	5	7	9	3	9
M19	12	3	3	3	3	0
M20	9	0	3	3	3	0
M21	35	5	7	9	5	9
M22	3	0	0	3	0	0
M23	33	5	7	9	3	9
M24	33	5	7	9	3	9
**Mean**	**15.2**	**2.4**	**3**	**4.4**	**1.9**	**3.6**

**Table A4:** Nutrition Quality.

Company	Total	Product Formulation	Marketing to All Consumers	Marketing to Children	Product Accessibility	Staff Health and Wellness	Consumer Programmes	Product Labelling
**Benchmark**	**25**	**5**	**3**	**3**	**5**	**3**	**3**	**3**
M1	9	3	0	0	3	1	1	1
M2	7	3	0	1	0	1	1	1
M3	18	5	0	1	3	3	5	1
M4	14	5	0	3	3	1	1	1
M5	5	3	0	0	0	1	1	0
M6	6	3	0	0	0	1	1	1
M7	7	3	1	0	0	1	1	1
M8	8	3	0	0	0	1	3	1
M9	8	3	0	0	3	1	1	0
M10	16	5	3	0	3	1	3	1
M12	16	5	3	5	0	1	1	1
M14	11	5	0	3	0	1	1	1
M16	8	3	0	0	0	1	3	1
M18	27	5	3	5	5	3	3	5
M19	13	5	0	1	3	0	3	1
M20	16	5	0	0	0	3	3	5
M21	28	9	5	5	0	3	3	3
M22	8	3	0	0	0	3	1	1
M23	27	9	5	5	3	3	1	1
M24	24	9	5	5	0	1	3	1
**Mean**	**13.8**	**4.7**	**1.3**	**1.7**	**1.3**	**1.6**	**2**	**1.3**
